# Evaluation of the social and economic impact of extreme weather events in people living with HIV in KwaZulu-Natal, South Africa (S3E project): Protocol for a mixed-method study

**DOI:** 10.1371/journal.pone.0325490

**Published:** 2025-07-09

**Authors:** Saeideh Babashahi, Kingsley Orievulu, Ekeminiabasi Eyita-Okon, Dominic Kniveton, Collins Iwuji

**Affiliations:** 1 Department of Global Health and Infection, Brighton and Sussex Medical School, University of Brighton and University of Sussex, Brighton, United Kingdom; 2 Africa Health Research Institute, Durban, South Africa; 3 Wits School of Governance, University of Witwatersrand, Johannesburg, South Africa; 4 School of Global Studies, University of Sussex, Brighton, United Kingdom; PLOS: Public Library of Science, UNITED KINGDOM OF GREAT BRITAIN AND NORTHERN IRELAND

## Abstract

The impacts of extreme weather events (EWEs), such as droughts and floods, continue to be felt inequitably and disproportionately, with vulnerable communities experiencing diverse effects. While more is known about the direct impacts of EWEs on the general population, there is limited quantified evidence regarding the indirect socioeconomic impacts of climate-driven EWEs on vulnerable sub-populations such as people living with HIV (PLHIV). Globally, South Africa has the largest number of PLHIV, who by the nature of their medical needs and the stigma around their disease burden, have different vulnerability profiles to the wider population. KwaZulu-Natal province, which recorded the second-highest burden of HIV in South Africa, has been hit hard by recurrent floods in recent years. This study aims to evaluate the social and economic impacts of recent floods on PLHIV in the province and co-develop a priority list of policy interventions that would address and minimise flood impacts among PLHIV. This study involves three objectives, and a combination of qualitative thematic analysis and economic assessment will be applied to investigate the socioeconomic impacts of the recent floods in PLHIV across two rural districts in South Africa. The study will apply a mixed-methods exposed-unexposed approach to assess the impacts of recent floods on PLHIV concerning treatment adherence, healthcare use, out-of-pocket expenses, coping mechanisms, work productivity loss, and other economic losses. An equity impact analysis will be conducted to examine how the socioeconomic impacts of floods are distributed disproportionately within the study sample. A priority list of policy interventions will be co-developed to minimise the impacts of floods using participatory research methods and multi-criteria decision analysis. Using a novel mixed-methods approach, this study contributes to understanding the social and economic impacts of recent floods on PLHIV and co-produces a list of adaptive and mitigation policy interventions to address these impacts.

## Introduction

Climate change is one of the most pressing public health issues that threatens to further widen health, social and economic disparities across and within societies [1–6]. The frequency, intensity and duration of extreme weather events (EWEs), such as droughts, floods, and cold and heat waves, have increased in the last few decades globally [3,4,7,8]. Only in 2024, many parts of the world experienced widespread EWEs, such as extreme heatwaves in the Sahel region of Africa [9], heavy rainfalls and flash floods in Germany and Spain [10,11], cold and heat waves in America [12] and tropical cyclone and floods in southeast Asia [13].

The World Meteorological Organization reported a sevenfold increase in disaster losses from EWEs since the 1970s, with the losses expected to be higher in lower-income countries and regions [14]. The impacts of climate-driven extreme events are extensive and include injuries, premature deaths, the spread of infectious disease, mental health impacts, displacement, disrupted healthcare access, food insecurity, water crisis, and disrupted social and economic stability [3,4,15,16]. Economic losses attributable to EWEs can cause livelihood insecurity, making affected populations reprioritise their livelihood needs. For people with pre-existing health conditions, including people living with HIV (PLHIV), it can eventually result in treatment nonadherence and poorer health outcomes, in addition to other issues such as displacement and poverty [4,7,17].

Climate-driven extreme events and their catastrophic consequences will continue to be distributed inequitably, with vulnerable communities and regions affected diversely [18,19]. While more is known about the consequences of climate-related extreme events on the general population, less attention has been paid to the pathways from climate hazards to poor health outcomes or socioeconomic insecurities for specific population sub-groups [5,14,20]. Vulnerable groups of the population, i.e., groups with characteristics susceptible to EWEs impacts [14,21], include older adults, children, and people with pre-existing health conditions. These sub-populations experience a disproportionate share of EWEs impacts, particularly in low and middle-income countries [20,22–24]. Climate change vulnerabilities are more pronounced across socio-economically marginalised communities and can be linked to gender disparities [22,23,25–27].

There is a dearth of evidence on the social and economic impacts attributable to EWEs (e.g., interruptions to healthcare and productivity losses) across vulnerable groups with pre-existing health conditions, such as people living with HIV (PLHIV), particularly in sub-Saharan Africa [5,14,20]. Like any other part of the world, the impact of climate change poses a significant threat to South Africa, and it is rapidly accelerating [18,28]. The April 2022 floods in KwaZulu-Natal (KZN), South Africa, resulted in more than 400 deaths, severe damage to infrastructure and disruption of services, including healthcare, with over 40,000 people displaced [29,30]. The province was again hit by more storms and flash floods in 2024, which caused further damages to infrastructure, including an estimated number of over 814 households (around 4,297 people) affected and 21 fatalities confirmed in the province [31]. South Africa currently ranks first globally in HIV cases, with an estimated 7.8 million PLHIV accounting for 13% of global HIV cases [32–34], and KZN is widely recognised as the epicentre of the HIV epidemic in South Africa, with an adult HIV prevalence of 30% [35].

Prior studies have investigated the impact of climate change and EWEs on lives and livelihood vulnerabilities using qualitative research approaches [24,25,36,37]. While qualitative research is imperative to reveal and elucidate the EWEs’ hidden impacts and pathways in more breadth and depth [37], quantitative data (e.g., productivity losses or healthcare costs) demonstrates the extent of impacts and allows for assessment of population subgroup differences [38]. There is clearly a need for both quantitative and qualitative methodologies to elucidate the complex, interrelated and multi-dimensional impacts of EWEs on humans.

The S3E study aims to explore and evaluate the economic, social, and health impacts of the recent KZN floods on PLHIV and co-create priority policy interventions to mitigate these impacts. The objectives of the study are to:

1. Understand the impact of floods on healthcare utilisation, lives and livelihoods amongst PLHIV.
2. Measure economic productivity losses and healthcare costs associated with recent floods in PLHIV.
3. Identify and create a priority list of sustainable and adaptive actions to minimise the health, economic and social impacts of floods on PLHIV.

## Materials and methods

### Study design and setting

The S3E study adopts a mixed-methods design where a mix of qualitative and quantitative data collection techniques will be applied to investigate experiences of PLHIV concerning disrupted access to healthcare, treatment adherence, and coping mechanisms in addition to economic losses (including productivity losses) during the recent floods in South Africa. A retrospective community-based exposed-unexposed approach will be carried out in relation to the first two study objectives.

**Fig 1:**
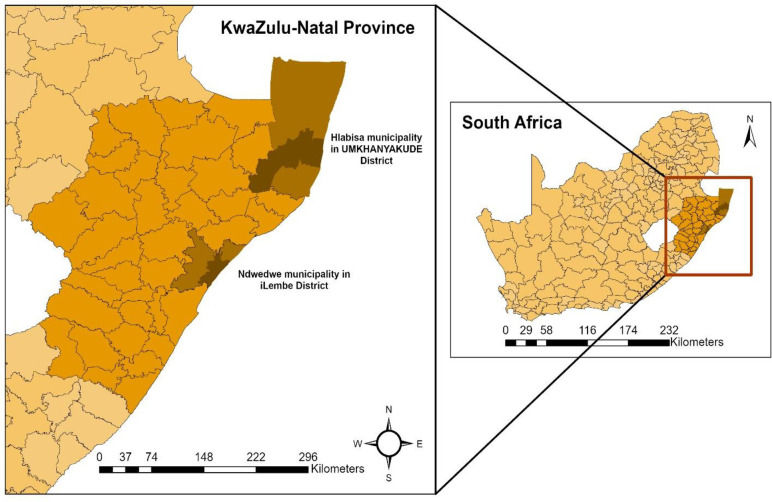
The map of the two study areas in KwaZulu-Natal province in South Africa. The white polygon in the South Africa map shows the Lesotho country. The map was created using ArcGIS Pro software version 3.4.0 supported by Esri. The basemap (e.g., map data and shapefiles) were derived from the United Nations Office for the Coordination of Humanitarian Affairs, Humanitarian Data Exchange (https://data.humdata.org/dataset/cod-ab-zaf).

The study will be implemented in the Hlabisa municipality, where the Africa Health Research Institute (AHRI) hosting the study is located, and the Ndwedwe municipality, iLembe district, both in KZN, South Africa. Fig 1 represents the map of the two study areas. These two municipalities were selected because Hlabisa (i.e., unexposed group) was relatively spared by the floods, whilst Ndwedwe, iLembe (i.e., exposed group) was the second most affected sub-district after the city of Durban. The two sub-districts have been selected for comparison as they have similar sociodemographic profiles; both areas are under-resourced rural and socio-economically disadvantaged, and most people live below the poverty line [39,40]. In both areas, HIV is the leading cause of death in individuals aged 25–64 years [41], and antenatal HIV prevalence exceeds 40% [42].

More detailed information concerning the study design for each study objective is provided in the following paragraphs.

#### Objective 1. Understanding the impact of floods on healthcare utilisation, lives and livelihoods (March 2025–September 2025).

To understand the impacts of the floods on PLHIV, we will conduct semi-structured in-depth interviews (IDIs) and focus group discussions (FGDs) across the two study sites. Interview questions will cover topics around how PLHIV make sense of their experience of the April 2022 KZN floods – especially for the unexposed population, while the unexposed population (in Somkhele) will explore participants’ views about the 2022 KZN floods and other floods and EWEs experienced in their contexts. The interviews and FGDs will explore how PLHIV were impacted by the floods, including their health, healthcare access and utilisation and livelihood sustenance. We will also interview key informants from health and health-determining sectors, including the Department of Health, Department of Social Development, and Disaster Management Agency, to gain an understanding of actions or inactions towards mitigating the impact of the floods.

#### Objective 2. Measuring the economic losses and healthcare related costs (March 2025–December 2025).

We will follow the recommended methods to identify and evaluate the impacts of socioeconomic factors associated with floods and measure the economic impacts and losses, including healthcare utilisation and related costs, attributable to recent floods for a randomly selected sample of PLHIV. The study design, analytical methods and reporting of the findings will follow the Consolidated Health Economic Evaluation Reporting Standards (CHEERS) Statement [43].

We will evaluate the economic burdens of floods in terms of direct and indirect costs (e.g., healthcare resources utilisation and work productivity loss). Individual-level data will be collected via structured questionnaires. A micro-costing approach (including a bottom-up construction of costs) will be employed to estimate costs associated with healthcare utilisation and other economic impacts of floods. The overall costs incurred due to floods will be estimated from a provider perspective (and a patient perspective, where data permits). We also collect socioeconomic and demographic information (e.g., age, gender, employment status, etc) of the study participants.

#### Objective 3. Co-development and co-prioritisation of policy actions (May 2025–May 2026).

In this objective, we will adopt a mixed-methods approach involving both quantitative and qualitative data collected through existing data sources, as well as engagements and workshops with key stakeholders involved in the care of PLHIV and those directly affected by the floods. This objective will be achieved through two main phases, as described below.

Phase 1: To identify early adaptive policy actions to minimise the health, economic and social impacts of floods on PLHIV

Phase 2: To create a priority list of early actions identified in Phase 1.

In phase 1, we will use the adapted Participatory Impact Pathways Analysis (PIPA) approach developed by the University of Sussex following the STEPS Centre [44,45]. PIPA provides a ‘road map’ that supports the co-development of a list of policy interventions by consulting and including multiple key stakeholders (e.g., patient representative, policymaker, health provider, etc). This approach is designed for complex contexts and provides a framework for ‘action research’ on processes of change. It has been successfully applied for co-production purposes in climate change research [45,46]. PIPA is a planning and, monitoring and evaluation tool designed to assist the people involved in a project, programme or organisation and make explicit their theories of change (i.e., how they see themselves achieving their goals and having an impact). Climate risk narratives are stories describing a subset of plausible – yet certainly not definitive – futures within the spread of climate projections intertwined with local context, impacts and vulnerabilities. Climate risk narratives enable the input of multi-model future climate scenarios into the project for exploring the health risks of future floods in PLHIV.

In phase 2, in line with the World Health Organization’s good practice for priority-setting [47–50], a multi-criteria decision analysis (MCDA)-based framework will be developed and implemented to rate, rank and prioritise the policy actions identified in phase 1 to support evidence-based climate change adaptation policymaking (particularly for flooding) involving key stakeholders and partners from all relevant sectors mentioned above via a workshop.

### Ethics and consent to participate

Ethical approvals for this study have been obtained from the University of Sussex, Brighton and Sussex Medical School Research Governance Ethics Committee (ER/BSMS9P2V/2/1) in the UK and the University of Kwazulu-Natal Biomedical Research Ethics Committee (BREC/00006821/2024) in South Africa. Participation in the study will be voluntary. All participants will sign a written informed consent form for participation and be given information that participation is voluntary and that they can withdraw from the study at any time.

### Overview of participant recruitment and inclusion criteria

We will recruit PLHIV in the Hlabisa subdistrict through a randomisation process. Potential participants will be randomly drawn from – and recruited – through the AHRI Health and Demographic Surveillance System (HDSS) [39]. In this programme, triannual household-based surveys are used to collect sociodemographic data from approximately 150,000 people in an 845km^2^ area coupled with annual home-based, point-of-care HIV testing, linked to real-time HIV clinical data and health service utilisation records from all 11 regional primary healthcare clinics that provide HIV care, hospitalisation data at the single district referral hospital, and annually death data carried out through verbal autopsies of all deaths in the catchment area. This research database will be used as a sampling frame to randomly select and invite PLHIV to take part in the study in Hlabisa.

AHRI does not have a similar research infrastructure in Ndwedwe, iLembe district. To identify PLHIV in the community without stigmatising them, we will hold monthly meetings with the community engagement department (CED) at AHRI to engage the health facilities and traditional structures in Ndwedwe as part of the community entry process, trust building and stakeholder engagement. Part of this process will entail a series of road shows and edutainment programmes – led by the CED – encapsulating games and educational talks on climate change and health to sensitise the community about the study. Subsequently, the study team will work closely with community caregivers and community-based organisations who have access to PLHIV to facilitate recruitment. Individuals who are recruited through this process will be informed about the study and offered participation if they meet the eligibility criteria. Although it is logistically easier to recruit PLHIV from healthcare facilities, this will introduce selection bias as it means only PLHIV who are still able to access healthcare facilities will be offered participation in the study, and these individuals may differ from the general population of PLHIV in Ndwedwe.

Participants will be recruited if (a) they are aged 18 years and over, (b) they were diagnosed with HIV before the April 2022 floods, (c) were on antiretroviral therapy before April 2022, (d) were resident in Ndwedwe (exposed group) or Hlabisa (unexposed group) over a month before and during April 2022, (e) were present for and remember the April 2022 floods (for the exposed group), and (f) they are able to give written consent.

More detailed data regarding the study participants and sample size for each study objective is provided in the following sections.

### Sample size

#### Objective 1. Understanding the impacts of recent floods.

Under objective 1, we will recruit a minimum of 30 participants (PLHIV) for the IDIs across the exposed and unexposed sites: about 15 participants per site to provide in-depth reflections on the floods and its impacts. We will also organise two FDGs – one in each site – consisting of about six to eight participants per group. We will aim to achieve age and gender balance in the selection of participants while taking into consideration the context-specific issues in each study site. Finally, we will recruit about ten key informants comprising policymakers from health, disaster management, social welfare and housing for the interview through the personal and professional networks of the research team. The actual number of IDIs and KIIs to be undertaken will depend on data saturation.

#### Objective 2. Measuring the economic losses and healthcare related costs.

The background loss from care after 12 months of antiretroviral treatment ranges from 20–30% [51]. Therefore, we will need to recruit a total of 424 PLHIV (212 unexposed and 212 exposed individuals) at a matched exposed-unexposed ratio of 1:1 (one unexposed for one exposed) to reach an 80% power at 0.05 significance level to detect a 90% increase in odds of loss from care during the first 6 months following the floods. Although we do not aim to estimate loss from care (i.e., loss from follow-up) in this study, it underpins our study hypothesis while not the primary reason for this study. The AHRI HDSS databases described in the participant recruitment section will serve as the sampling frame for randomly selecting the 212 unexposed individuals in Hlabisa, of whom 70% will be female, which is representative of the proportion of females amongst all PLHIV who engage with the surveillance programme. A similar approach will be taken to recruit 212 exposed PLHIV in Ndwedwe by working closely with AHRI CED, community caregivers and other community-based organisations, as mentioned above.

#### Objective 3. Co-development and co-prioritisation of policy actions.

In this objective, we will engage a minimum of at least 15–20 stakeholders, including policymakers in health and health-determining sectors (water, housing, energy), civil society and advocacy groups, healthcare providers, PLHIV, and health system researchers who will be invited through the personal and professional networks of the research team in the workshops in both phases of the objective.

### Patient and public involvement

PLHIV and public advocates will be involved at every level and phase (research preparation, execution and knowledge translation) of this study. The study team will work closely with community caregivers and community-based organisations who have access to PLHIV to facilitate recruitment.

### Data collection

Study materials, including participant information sheets, written consent forms, and questionnaires, will be translated into isiZulu, the local language, by trained supervised research assistants proficient in English and Zulu. Questionnaires and other study materials will be made available in either English or Zulu at the participant’s request.

#### Objective 1. Understanding the impacts of floods.

IDIs and KIIs are expected to last between 45 minutes and 1 hour. The FGDs will also last between one to two hours. The duration of these interviews often depends on the disposition of participants and their willingness to actively engage (or not). It thus requires the interviewers’ ability to communicate and engage effectively with the participants. Consequently, data collection under this objective will be undertaken by experienced social science researchers.

All interviews will be audio-recorded, transcribed verbatim and translated to the closest English meaning by experienced social science researchers. Where a participant refuses to be audio-recorded for the interviews, the interviewer(s) will take adequate notes – mental and physical – to help with the development of a comprehensive summary narrative that would suffice in lieu of the interview transcript [52]. The note will endeavour to comprehensively capture what was said by the participant(s). These transcripts will be quality controlled by trained social science study coordinators to ensure their quality for data analysis. The data collection for this objective commenced on the 4^th^ of April 2025 in Hlabisa (the unexposed study area). All recruited participants provided written informed consent, which was documented by the local research team.

#### Objective 2. Measuring the economic losses and healthcare related costs.

The primary data for this objective will be collected after the preliminary analysis of qualitative data collected for objective 1, which will feed back into reviewing and finalising the questionnaires for objective 2. Study questionnaires will then be pilot-tested with a small portion of the study sample to identify potential issues and assess the rigour of questionnaires. The findings of the pilot study will be analysed to inform, test and revise our econometric model. The questionnaire for this objective has been initially developed by the research team (which includes a clinician, climate scientist, social scientist, health economist and policy analyst) and was then pre-tested by interviewing six researchers from a variety of backgrounds (e.g., ethics, anthropology, global health sciences, nursing, economics), as well as two operational managers from Somkhele and Ndwedwe community health clinics and two research assistants experienced in working with PLHIV in rural South Africa. This allowed to check for clarity, understandability and relevance of the questions and revise the questionnaire. The pre-tested questionnaire is expected to take 60–90 minutes to complete.

Trained supervised research assistants will facilitate and make appointments with participants to complete questionnaires. Face-to-face interviews will be carried out one by one with a research assistant in one of AHRI Somkhele office’s rooms (for the unexposed group) and the Ndwedwe Community Health Centre or a participant-researcher mutually agreed location (for the exposed group) who will use a tablet to enter data into the Research Electronic Data Capture (REDCap), a secure web-based platform widely used for data collection and management for this objective. The coordinating team at AHRI will export data from REDCap for data curation and cleansing.

#### Objective 3. Co-development and co-prioritisation of policy actions.

Phase 1 involves using the core elements of PIPA that include the problem tree analysis; visioning exercises; current and future network maps, indicating interactions and the linkages required to achieve project aims; and the ‘outcomes logic model’ [53,54]. Different groups of key stakeholders will be involved, and the PIPA approach will be employed through a workshop in one of AHRI offices [44,54] for their framings of how they see PLHIV and their healthcare being affected by floods, now and into the future. This follows the recognition that who you are shapes how you frame or understand a system or a problem. The information from this phase will be used to identify adaptation early action interventions. For instance, PLHIV, a local health clinic worker, a hospital doctor, a climate scientist, a national health minister, and a multinational pharmaceutical firm might all frame the climate issues affecting PLHIV in different ways. Those various framings will lead to different narratives being told about the same system and different choices being made. Too often the narratives of powerful actors and institutions become the motorways channelling policy, governance and interventions, overrunning the valuable pathways responding to poorer people’s own goals, knowledge and values.

The pathways approach examines multiple pathways and backed by a variety of practical methods that opens up space for more plural and dynamic healthcare resilience [44,45]. It aims to open up the political process of building pathways that are currently hidden, obscured or oppressed. Specific practical methods used in this project will include system mapping of climate-health links that are relevant to PLHIV, PIPA and climate risk narratives [55]. Systems mapping of climate-health links helps make explicit and visualise the causal relationships and pathways from floods to poor livelihood and healthcare outcomes [56].

Phase 2 of this objective also involves two other workshops to be held in the AHRI offices, engaging the same groups of key stakeholders mentioned above in phase 1. In workshop 1, an overarching set of prioritisation criteria will be specified through a brainstorming and think-aloud session of stakeholders, including PLHIV and public advocates. Workshop 2 involves the priority-setting exercise, where actions identified in phase 1 will be co-rated, co-ranked and co-prioritised based on criteria finalised in workshop 1. The priority list of actions will be simplified into three tiers of priority: i.e., ‘high urgency’, ‘medium urgency’ and ‘low urgency’ for ease of communication (e.g., with researchers and policymakers) [57].

### Data management

All data from the three objectives will be stored on password-protected computers at AHRI, South Africa, and only authorised personnel will have access to them. Research data will only be identifiable by Participant ID numbers (PIDs), which will be assigned at the time of consent. The document linking participants to their PID number will be kept separately in a secure place and will be anonymised in line with the Standard of Operation used at AHRI Social Science Core. Access to data will be controlled with user IDs and secure password protection and multi-level rights that grant different privileges to different members of the research team.

Both AHRI and the University of Sussex act as data controllers. However, all personal data (i.e., names and contact details) will be kept in AHRI, South Africa, and not transferred to the UK. Research data (including potentially identifiable data, e.g., demographic information) transferred to the University of Sussex will be pseudonymised and processed in line with the UK General Data Protection Regulation (GDPR) 2016. Pseudonymised data will also be stored electronically and securely at Sussex and Brighton Medical School OneDrive and SharePoint clouds and backed up at the University of Sussex secure repository. Unidentifiable data will be kept as part of the study and will be made available upon request after publishing the study findings.

### Data analysis

#### Objective 1. Understanding the impact of recent floods.

We will analyse the data from the IDIs, KIIs and FGDs that have been transcribed, fully translated and quality-controlled using the thematic analysis approach [58,59]. Field notes and or interview summaries will also serve as data sources with the translated transcripts to give a full picture of the data from the purview of the interviewer(s)/researchers. The data will be managed through the NVivo data analysis software, and the coding process will be a mix of inductive and deductive thematic coding. We will however also carry out an initial open coding of individual manuscripts to ascertain general codes emerging within the data. This will then be followed by organising them into themes and subthemes based on some pre-identified categories linked to the social, economic, health and mental/psychosocial impacts of floods, including adaptation and mitigation strategies. Field notes and thematic summaries will be developed and prepared on the key topics as they emerge from the interviews. Preliminary findings will be drawn from summaries to aid in the preparation of briefing notes to disseminate these findings among our stakeholders.

#### Objective 2. Measuring the economic losses and healthcare related costs.

Following the recommended methods, we will construct a multi-variate econometric model to identify, understand and measure the impact and extent of socioeconomic impacts of recent floods across PLHIV, adjusting for baseline covariates such as age, gender, etc. We will also conduct a cost analysis to measure the economic burdens of floods in terms of direct and indirect costs in South African Rand (SAR), local currency. The SAR will be then converted to US dollar (USD) using the purchasing power parity (PPP) conversion factor recommended by the World Bank [43,60]. The economic impacts of the floods, in terms of the loss of work productivity, will be estimated using the human capital approach to reflect the value of lost production due to absenteeism and presentism associated with recent floods for PLHIV [61,62]. Both descriptive and inferential statistical analysis will be used for comparison purposes, as well as sub-group analysis across exposed and unexposed groups. Following recommended methods, multiple imputations will be used to deal with missing data [63–65]. We will calculate confidence intervals and undertake simulation, bootstrapping and sensitivity analysis to map and assess the uncertainty in our parameter estimates and the robustness of our findings. We will also undertake an equity impact analysis through subgroup analyses of the economic impacts of floods linked with the socioeconomic and demographic characteristics (e.g., age, gender, wealth quintile, etc) of the study sample across exposed and unexposed groups. Data analysis will be undertaken in STATA 16.

#### Objective 3. Co-development and co-prioritisation of policy actions.

After the Phase 1 workshop, the facilitators will use the workshop outputs to synthesise the outputs qualitatively and quantitatively, prepare narratives including the underlying logics and assumptions and construct the outcomes logic model and the network maps. The workshop outputs will be visualised using MS Office tools or one of the social analysis software available for free [43–45].

Phase 2 consists of developing a MCDA-based framework in MS Excel for prioritising policy actions and interventions that will involve the following three key components: (i) identifying policy actions (output of phase 1) to be prioritised; (ii) specifying prioritisation criteria (i.e., the characteristics or attributes of actions); and (iii) measuring weights for the criteria, representing their relative importance to stakeholders (e.g., patient and public advocates, researchers, policymakers, etc) [49,50]. These components will be applied to co-rate each policy action according to its ‘performance’ on each criterion. Finally, the ratings will be aggregated using a weighted sum model – to co-produce a total score (typically in the range of 0–100%) for each action [49,50,57,66], by which the actions are ranked (and prioritised). A sensitivity analysis will be performed to check the robustness of the results.

### Policy analysis (May 2025–August 2026)

The existing policy documents such as the National Climate Change Response and Climate Change Act 22 of 2024, etc will be thematically analysed to deduce themes capturing the climate change-health nexus, with a particular focus on PLHIV. Drawing on the findings of Phases 1 and 2 in objective 3 as well as review of national policy documents, we will conduct a gap analysis. This will entail a comparative assessment of the current/existing state of climate-health (HIV) policy with the outcomes of Phases 1 and 2. We will embark on gap identification – to deduce areas of policy divergence and convergence – as well as gap prioritisation, against the backdrop of changing climatic conditions (with priority on floods) in KZN/South Africa. This analysis will inform evidence-based policy recommendations for key stakeholders in South Africa.

## Discussion

The present study aims to evaluate and understand the social and economic impacts (including coping strategies, access to care, healthcare service utilisation and associated costs, work productivity losses and other socioeconomic instabilities) of the recent floods among PLHIV in rural South Africa and use participatory research methods and MCDA to co-design and co-rank a list of policy action interventions. To the best of our knowledge, this study is the first study to use this particular mixed-methods approach to provide an in-depth analysis of the social and economic pathways for how climate-induced extreme events, such as floods, are likely to affect vulnerable communities such as PLHIV.

South Africa is prone to climate-driven EWEs. In 2015, KZN experienced one of the worst droughts recorded in the country, which resulted in the declaration of a state of disaster [67]. Orievulu et al. (2022) investigated the impact of drought on HIV treatment and care elucidating mechanistic pathways that implicated food insecurity and productivity loss in the context of pervasive poverty that was gendered [25,68]. The April 2022 floods in KZN, also the province most impacted during the 2015 droughts, resulted in hundreds of deaths, damage to infrastructure and disruption of services, including healthcare. Over 40,000 people were displaced because of the floods, many of whom are still homeless [29,69]. KZN is the epicentre of the HIV epidemic in South Africa, with more than one-third of the adult population living with HIV [39]. The impact of EWEs, such as floods, can result in acute interruption of treatment and care in PLHIV [25,68], and competing livelihood priorities could mean that some of these individuals may not return to care, resulting in chronic disengagement and associated increase in morbidity, loss of income and mortality.

Our study will have several important implications. Findings from the S3E project will provide new insights into the disproportionate impacts of floods on the health, livelihoods and well-being of PLHIV in rural South Africa. This study will improve our knowledge on the extent of the economic losses and disrupted access to and use of healthcare due to recent floods as well as the coping mechanisms across rural vulnerable populations, particularly PLHIV. Our research is intended to generate long-term co-benefits at individual, community and policy levels by involving main groups of key stakeholders (patient and public advocates, researchers, policymakers) to co-produce and co-prioritise a list of adaptation interventions to inform policy and practice on which interventions can address the multi-faceted impacts of EWEs such as floods.

We will develop policy briefs and prepare and make verbal presentations of preliminary and final findings at the AHRI Indaba series (webinars) and symposia. We will also present our findings to the local communities through community road shows, newsletters written in the local language and radio programmes. The study findings will also be disseminated in high-impact journals and national and international conferences.

## Supporting information

S1 Appendix Inclusivity in global research questionnaire. (PDF)
